# Trends of antimicrobial susceptibilities and multidrug-resistant colonization rate in patients transferred from long-term care facilities during 2017–2022: a cross-sectional study

**DOI:** 10.1186/s12879-024-09145-y

**Published:** 2024-02-21

**Authors:** Jin Ju Park, Hyejin Park, Sun Hee Na, Yu Bin Seo, Jacob Lee

**Affiliations:** Division of Infectious Disease, Department of Internal Medicine, Kangnam Sacred Heart Hospital, Hallym University College of Medicine, 1 Singil-Ro, Yeongdeungpo-Gu, Seoul, 07441 Republic of Korea

**Keywords:** Long-term care facilities, Antibiotic resistance, Carbapenem-resistant *Enterobacterales*

## Abstract

**Background:**

With the global increase in the older population, the proportion of those receiving care in long-term care facilities (LTCFs) has also been increasing. We assessed the epidemiology, antibiotic susceptibility, and colonization status of drug-resistant organisms in patients transferred from LTCFs.

**Methods:**

We retrospectively reviewed the medical records of patients transferred from LTCFs between 2017 and 2022. The reasons for admission, antimicrobial susceptibility, and colonization rates of carbapenem-resistant *Enterobacterales* (CRE), methicillin-resistant *Staphylococcus aureus* (MRSA), and carbapenem-resistant *Acinetobacter baumannii* (CRAB) were recorded. We analyzed the susceptibility and colonization rates by year to identify trends.

**Results:**

Of the 936 patients transferred from LTCFs, 54.3% were admitted to the intensive care unit and 12.5% died. The most common reason for admission was infection (*n* = 573, 61.2%), followed by gastrointestinal bleeding (*n* = 67, 7.2%) and cerebrovascular disorder (*n* = 65, 6.9%). A total of 452 *Enterobacterales* strains were isolated, and their susceptibility rates to ciprofloxacin and cefotaxime were 33.3% and 35.6%, respectively. A total of 54.9% were extended-spectrum beta-lactamase-producing strains, and 4.9% of them were carbapenem-resistant, both of which showed an increasing trend (*P* = 0.024 and *P* < 0.001, respectively). The prevalence rates of CRE, CRAB, and MRSA colonization were 9.2%, 7.1%, and 23.1%, respectively. CRE colonization showed a significant increase (*P* < 0.001), with carbapenemase-producing *Enterobacterales* accounting for 75.9% of cases.

**Conclusions:**

Patients transferred from LTCFs are primarily affected by infections and exhibit high resistance rates. The increasing trend in CRE colonization rates each year highlights the need for the implementation of rigorous infection control measures for effective management.

**Supplementary Information:**

The online version contains supplementary material available at 10.1186/s12879-024-09145-y.

## Background

Many countries worldwide have entered an era of an aging society and are making efforts to address the needs of their older populations [1]. Long-term care facilities (LTCFs) are institutions where individuals who require assistance, such as medical care or rehabilitation therapy, because of old age or underlying health conditions, can receive the necessary care. In Korea, more than 18.5% of the total population is aged 65 years or older, marking the onset of an aging society [2]. It is estimated that by 2050, approximately 45% of the total population will be aged 65 or older [2]. Consequently, the number of individuals using LTCFs is anticipated to increase annually.

One of the main causes of transfer from LTCFs to acute care hospitals is infection [3]. Patients transferred from LTCFs often have compromised immune systems owing to age or underlying health conditions, making the early use of antibiotics particularly crucial for prognosis [4]. Access to antibiogram data is essential to ensure the appropriate early use of antibiotics. In domestic LTCFs, antibiograms for all admitted patients are limited; therefore, regular updates are required.

LTCFs can serve as reservoirs for multidrug-resistant organisms, particularly carbapenem-resistant *Enterobacterales* (CRE) [5]. Surveillance studies conducted on patients transferred from LTCFs in South Korea between 2016 and 2017 revealed a low prevalence of CRE, with a rate of only 1.4%; none of these cases involved carbapenemase-producing *Enterobacterales* (CPE) [6]. Additionally, in a study conducted during 2018–2019, there were no cases of CRE [7]. Given the global increase in multidrug-resistant organisms, it may be necessary to assess changes in the patterns of CRE colonization and develop plans for selective screening of patients transferred from LTCFs to establish appropriate infection control measures [8–10].

In this study, we aimed to assess the epidemiology and antibiotic susceptibility of patients transferred from LTCFs. Additionally, we analyzed the colonization status of drug-resistant organisms. Based on these results, we aimed to understand the fundamental characteristics of patients transferred from LTCFs, establish a rationale for the appropriate selection of initial antibiotics for infections, and provide a foundation for infection control measures.

## Materials and methods

### Study design and population

This study was conducted from 2017 to 2022 at a 573-bed university hospital in South Korea. To assess the epidemiology and antibiotic susceptibility of patients transferred from LTCFs, we retrospectively reviewed the medical records of patients transferred from LTCFs. This study included cases in which patients were transferred from LTCFs to the main hospital via the satellite emergency department, whereas those admitted from LTCFs owing to coronavirus disease (COVID-19) were excluded. Only the first admission was included in the analysis of the patients with multiple admissions.

### Data collection and data analysis

A retrospective analysis of the patients’ electronic medical records (EMRs) was conducted to review sex, age, and underlying medical conditions. The Charlson Comorbidity Index (CCI) was calculated. Reasons for admission, length of hospital stay, admission to the intensive care unit, and mortality were investigated. The reasons for admission, including possible infection, were validated by a physician based on a review of the admission records and clinical symptoms documented in the EMR.

The culture results of the patients at the time of admission were reviewed. Blood, sputum, urine, and other samples were collected. The decision regarding specimen collection and choice of specimen site for testing, carried out when infection was suspected, were also determined by the primary physician. In the case of coagulase-negative *Staphylococcus* species (CoNS), *Propionibacterium* spp., and *Corynebacterium* spp., only two or more subsequent blood cultures showing the same bacterial species with identical antibiograms were included in the analysis. The antibiotic sensitivity of the cultured strains was determined. For *Enterobacterales*, the presence of extended-spectrum beta-lactamases (ESBLs) and carbapenem resistance was also determined. Carbapenem resistance was determined based on resistance to any of the carbapenem antibiotics, including ertapenem, imipenem, and meropenem. In cases where *Proteus* spp., *Morganella* spp., and *Providencia* spp. exhibited intrinsic resistance to imipenem, they were considered resistant if they exhibited resistance to any other type of carbapenem besides imipenem. The antibiotic sensitivity of ESBL-producing *Enterobacterales* was further analyzed using subgroup analysis. Carbapenem resistance of *Pseudomonas aeruginosa* and *Acinetobacter baumannii* was also assessed. Methicillin resistance was determined for *Staphylococcus* species (*Staphylococcus aureus* and CoNS).

The institution recommended CRE colonization testing for patients transferred from LTCFs. Additionally, for patients admitted to the intensive care unit, colonization testing for carbapenem-resistant *A. baumannii* (CRAB) and methicillin-resistant *S. aureus* (MRSA) is performed. This study determined the results of CRE, MRSA, and CRAB colonization tests conducted within 48 h of admission.

An additional analysis was conducted of patients from whom CRE, CRAB, or MRSA species were cultured in clinical specimens to determine CRE, CRAB, or MRSA colonization status, respectively.

### Microbiologic data

The bacterial species were identified using the VITEK MS system (BioMérieux, Marcy-l’Etoile, France). The minimal inhibitory concentrations (MICs) of the antibiotics were determined using the VITEK 2 system (BioMérieux). The results were interpreted according to Clinical and Laboratory Standards Institute (CLSI) guidelines [11, 12]. Isolated microorganisms were classified as susceptible, intermediate-resistant, or resistant to certain antibiotics according to the CLSI recommendations. For *Enterobacterales* isolates nonsusceptible to carbapenem (based on an MIC of > 0.5 μg/mL for ertapenem or an MIC of > 1 μg/mL for imipenem or meropenem), the modified Hodge test and carbapenemase inhibition test were used for CPE detection.

### Statistical analysis

Categorical variables are presented as frequencies and proportions, whereas continuous variables are presented as medians (interquartile ranges [IQRs]). The chi-square and Fisher's exact tests were used to compare categorical variables. The trends in pathogens and their antimicrobial susceptibility according to year were analyzed using the Cochran–Armitage trend test. A *P-*value of < 0.05 was considered statistically significant. Statistical analyses were performed using R software (R Foundation for Statistical Computing, Vienna, Austria).

## Results

### Study population and clinical information

Throughout the study period, a total of 2,443 patients were transferred, among whom 1,326 originated from LTCFs. Patients with COVID-19 were excluded from the analysis, resulting in a final cohort of 1,110 patients. After removing duplicate cases, 936 patients were ultimately included in the analysis. The median age of the included patients was 79 (IQR: 71–85) years, and 404 (43.1%) patients were males (Table 1). The median CCI score was 5 (IQR: 4–6). When analyzing the reasons for admission, infection was the most common reason (*n* = 573, 61.2%), followed by gastrointestinal (GI) bleeding (*n* = 67, 7.2%), cerebrovascular disorder (*n* = 65, 6.9%), and orthopedic disorder (*n* = 43, 4.6%) (Table 1). Among these infections, pneumonia was the most frequent (*n* = 287, 50.1%), followed by urinary tract infection (UTI) (*n* = 173, 30.2%), GI infection (*n* = 31, 5.2%), and biliary tract infection (*n* = 28, 4.9%). Among the patients, 508 (54.3%) were admitted to the intensive care unit, and 117 (12.5%) died during hospitalization.

**Table 1:** Baseline and clinical characteristics of the study population (*N* = 936)

Characteristic	N	%
Age, years, median (IQR)	79 (71–85)	
Sex		
Male	404	43.1
Female	533	56.9
Comorbidity		
Hypertension	577	61.6
Cerebrovascular disease	411	43.9
Diabetes mellitus	330	35.2
Dementia	286	30.5
Cardiovascular disease	192	20.5
Chronic kidney disease	82	8.8
Solid tumor	82	8.8
Charlson’s comorbidity index, median (IQR)	5 (4–6)	
Reason for transfer		
Infection	573	61.2
Gastrointestinal bleeding	67	7.2
Cerebrovascular disorder	65	6.9
Orthopedic disorder	43	4.6
Renal disorder	34	3.6
Cardiogenic disorder	33	3.5
Gastrointestinal disorder	28	3.0
Source of infections (*n* = 573)^a^		
Pneumonia	287	50.1
Urinary tract infection	173	30.2
Gastrointestinal infection	31	5.4
Biliary tract infection	28	4.9
Skin and soft tissue infections	23	4.0
Length of stay, median (IQR)	13 (7–23)	
ICU admission	508	54.3
In hospital mortality	117	12.5

*IQR* Interquartile range, *ICU* Intensive care unit

^a^Source of infections further included *Clostridioides difficile* infection (*n* = 15, 2.6%), other lung infections such as influenza, lung abscess, and respiratory tuberculosis (*n* = 14, 2.4%), bone and joint infections (*n* = 8, 1.4%), catheter-related infections (*n* = 7, 1.2%), and other infections such as infective endocarditis and ear infections (*n* = 5, 0.9%)

### Isolated microorganisms according to specimen and antimicrobial susceptibility

Among the patients analyzed, blood, urine, and sputum cultures were obtained in 703 (75.1%), 688 (73.5%), and 412 (43.0%) patients, respectively. In the blood cultures, 123 (17.5%) yielded positive culture results, with 59 g-positive bacteria and 64 g-negative bacteria (Table 2). The most commonly cultured pathogen was *Escherichia coli* (*n* = 39, 31.7%), followed by CoNS (*n* = 32, 26.0%), *S. aureus* (*n* = 18, 14.6%), and *Klebsiella pneumoniae* (*n* = 9, 7.3%). In the urine cultures, 369 cultures (53.6%) showed bacterial growth, with *Enterobacterales* being predominant in 263 cases (71.3%). *E. coli* was the most commonly isolated bacterium (*n* = 159, 43.1%), followed by *Enterococcus* spp. (*n* = 42, 11.4%). Sputum cultures yielded bacterial growth in 229 cases (55.6%*). K. pneumoniae* was the most frequently isolated bacterium (*n* = 54, 23.6%), followed by *P. aeruginosa* (*n* = 50, 21.8%) and *A. baumannii* (*n* = 36, 15.7%).

**Table 2:** Isolated microorganisms according to specimens

Pathogen	Blood (*N* = 123) N (%)	Urine (*N* = 369) N (%)	Sputum (*N* = 229) N (%)	Others (*N* = 50) N (%)
*Enterobacterales*	62 (50.4)	263 (71.3)	105 (45.9)	22 (44.0)
*Escherichia coli*	39 (31.7)	159 (43.1)	20 (8.7)	9 (18.0)
*Klebsiella pneumoniae*	9 (7.3)	40 (10.8)	54 (23.6)	2 (4.0)
*Proteus mirabilis*	7 (5.7)	40 (10.8)	13 (5.7)	7 (14.0)
*Citrobacter species*	4 (3.3)			
*Providencia species*		9 (2.4)	5 (2.2)	
Other	3 (2.4)	15 (4.1)	13 (5.7)	4 (8.0)
*Coagulase-negative Staphylococcus species*	32 (26.0)	5 (1.4)		2 (4.0)
*Staphylococcus aureus*	18 (14.6)	2 (0.5)	32 (14.0)	8 (16.0)
*Streptococcus species*	6 (4.9)	6 (1.6)	6 (2.6)	2 (4.0)
*Enterococcus species*	3 (2.4)	42 (11.4)		4 (8.0)
*Pseudomonas species*	2 (1.6)	42 (11.4)	50 (21.8)	6 (12.0)
*Acinetobacter baumannii*		9 (2.4)	36 (15.7)	6 (12.0)

**Fig. 1:**
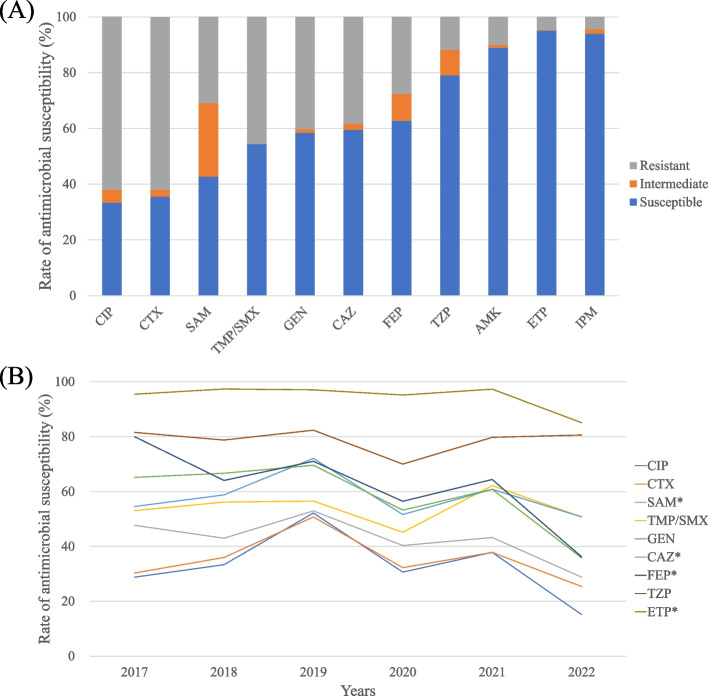
Antimicrobial susceptibility and ESBL-producing rate according to years in *Enterobacterales* isolates. **a** Overall antimicrobial susceptibilities and (**b**) trend of antimicrobial susceptibilities in *Enterobacterales* isolates from 2017 to 2022*.* Susceptibilities to ampicillin/sulbactam, ceftazidime, cefepime, and ertapenem significantly decreased according to years (*P* = 0.046, *P* < 0001, *P* < 0.001, and *P* = 0.013, respectively). Abbreviations: CIP, ciprofloxacin; CTX, cefotaxime; SAM, ampicillin/sulbactam; TMP/SMX, trimethoprim/sulfamethoxazole; GEN, gentamicin; CAZ, ceftazidime; FEP, cefepime; TZP, piperacillin/tazobactam; AMK, amikacin; ETP, ertapenem; IPM, imipenem; ESBL, extended spectrum b-lactamase

In total, 452 *Enterobacterales* isolates were obtained during the study period. The susceptibility rates of ciprofloxacin and cefotaxime against *Enterobacterales* were 33.3% and 35.6%, respectively (Fig. 1a–b). The susceptibility rates of cefepime and piperacillin/tazobactam were 62.7% and 79.0%, respectively, whereas that of ertapenem was 95.1%. Trend analysis of *Enterobacterales* susceptibility testing involving 11 antimicrobial agents revealed a significant decrease in susceptibility over the 6-year study period for 5 antimicrobials. Specifically, the susceptibility decreased from 47.7% to 36.2% (*P* = 0.046) for ampicillin/sulbactam, from 65.2% to 35.8% (*P* < 0.001) for ceftazidime, from 80.0% to 35.8% (*P* < 0.001) for cefepime, from 95.5% to 85.1% (*P* < 0.001) for ertapenem, and from 98.0% to 86.5% (*P* < 0.001) for imipenem. Carbapenem resistance was observed in 4.9% of cases The rate of carbapenem resistance in *Enterobacterales* increased significantly over the 6-year period, from 3.0% in 2017 to 14.9% in 2022 (*P* < 0.001). Carbapenem resistance varied according to the specimen type, with rates of 4.9% in blood cultures, 3.0% in urine cultures, and 7.6% in sputum cultures. Regarding ESBL results from the 426 samples, 54.9% (*n* = 234) were ESBL-producing strains at the time of admission. The proportion of ESBL-producing strains showed an increasing trend over the 6-year period, starting at 36.9% in 2017 and reaching 60.4% in 2022 (*P* = 0.024). The susceptibility rates of the ESBL-producing strains to various antibiotics were as follows: amikacin, 86.1%; piperacillin/tazobactam, 73.0%; sulfamethoxazole/trimethoprim, 44.5%; cefepime, 43.7%; ampicillin/sulbactam, 28.8%; and ciprofloxacin, 13.9%.

Among *P. aeruginosa* isolates, 52.0% exhibited susceptibility to ceftazidime, whereas 52.0% showed carbapenem resistance. Among *A. baumannii* isolates, 90.2% exhibited carbapenem resistance (See Supplementary Fig. 1, Additional File 1).

Of the 99 *S. aureus* and CoNS isolates, 78.8% were methicillin-resistant (MRSA or MR-CNS). The prevalence of methicillin resistance showed a decreasing trend (*P* = 0.005) (See Supplementary Fig. 1, Additional File 1).

### Results of active surveillance for colonization

CRE colonization testing was conducted in 855 (91.3%) patients, whereas colonization testing for CRAB and MRSA was conducted in 450 (48.1%) patients. The prevalence rates of CRE, CRAB, and MRSA colonization were 9.2%, 7.1%, and 23.1%, respectively (Table 3). When observed on a yearly basis, CRE colonization showed a significant increase, starting at 2.5% in 2017 and increasing to 26.1% in 2022 (*P* < 0.001). CPE accounted for 75.9% of CRE cases. The rates of CRAB and MRSA colonization did not show consistent trends over time (*P* = 0.189 and *P* = 0.969, respectively).

**Table 3:** Prevalence of drug-resistant organism colonization

	Overall	2017	2018	2019	2020	2021	2022	*P*-value
CRE	9.2	2.5	5.6	7.9	9.7	8.1	26.1	< 0.001
CRAB	7.1	6.9	6.5	3.0	13.6	5.0	13.7	0.189
MRSA	23.1	25.0	25.2	13.0	34.1	30.0	17.6	0.969

*CRE* Carbapenem-resistant *Enterobacterales*, *CRAB* Carbapenem-resistant *Acinetobacter baumannii*, *MRSA* Methicillin-resistant *Staphylococcus aureus*

### Colonization status in patients with clinically resistant pathogens

Among the 54 patients with both MRSA status and colonization results available, the colonization rate was significantly higher among patients with MRSA infections than among those with non-MRSA infections (59.1% vs. 40.9%, *P* = 0.001). Among the 207 patients with both CRE status and colonization results available, the colonization rate was significantly higher among patients with CRE infections cultured than among those with non-CRE infections (91.7% vs. 8.3%, *P* < 0.001). Among the 44 patients with both CRAB status and colonization results available, the colonization rate did not differ significantly among those with CRAB and non-CRAB infections (66.7% vs. 33.3%, *P* = 0.065).

## Discussion

In this study, we analyzed the epidemiology and antibiotic susceptibility of patients transferred from LTCFs. Globally, antibiotic resistance has become a substantial concern, and admission to LTCFs is recognized as a risk factor [13, 14]. In South Korea, there is a lack of data on antibiotic resistance rates among patients transferred from LTCFs; these data are crucial for appropriate antibiotic selection during initial treatment. Moreover, the increasing number of patients with antibiotic resistance highlights the importance of infection control of multidrug-resistant organisms in acute care medical facilities, emphasizing the need for up-to-date prevalence data.

In this study, the patients transferred from LTCFs had a median age of 79 years, indicating an older population. Their CCI scores were also high (5 points), signifying a high burden of underlying conditions. Given that 54.3% of the patients were admitted to the intensive care unit, those transferred from LTCFs had a higher severity level, with a mortality rate of 12.5%. The primary reasons for admission were infections, pneumonia, and UTI, which accounted for over 80% of all infections. Considering that patients from LTCFs, who are part of the high-risk group, are frequently admitted primarily because of infections, there should be increased emphasis on infection prevention in LTCFs. Although the complete prevention of infections in LTCFs may be challenging, it is crucial to implement preventive measures such as appropriate suction, catheter management, and infection control to reduce the risk of nosocomial infections in such settings.

It can be asserted that the selection of initial antibiotics for infections in high-risk groups is even more critical. Among patients transferred from LTCFs, *Enterobacterales*, with *E. coli* being the most prevalent, were commonly isolated. Cefotaxime susceptibility in *Enterobacterales* isolates was observed in only 35.6%, with over 50% being ESBL-producing strains. According to Kor-GLASS results, the resistance of *E. coli* to cefotaxime was 36.0% from 2017 to 2019 [15]. Kim et al. reported a susceptibility of 68.0% to cefotaxime in *E. coli*, and 33.1% of ESBL-producing strains were cultured from community-onset UTIs in 2017 [16]. Another study showed that in LTCFs, the resistance rate of *E. coli* to cefotaxime gradually increased, reaching 70% in 2018 [17]. When considering our results in conjunction with those of previous studies, it becomes apparent that the resistance rate and proportion of ESBL-producing strains in LTCFs are higher than those in the general population, and this trend is increasing.

*Enterobacterales* are the predominant causative pathogens of UTIs and intra-abdominal infections, with some isolates from sputum cultures. When dealing with infections from these sources, particularly in patients transferred from LTCFs, it is necessary to consider the possibility of ESBL-producing strains. Although carbapenems are the primary choice of antibiotics for ESBL-producing strains, piperacillin/tazobactam can be considered as an alternative in situations where carbapenem use is problematic owing to carbapenem resistance, given its susceptibility of over 70% against ESBL-producing strains. Although the clinical efficacy of piperacillin/tazobactam may vary, it can be a viable choice for treating UTIs when clinical improvement is observed [18–20]. Indeed, initial empirical treatment options, such as ciprofloxacin or trimethoprim/sulfamethoxazole, might have limitations or may not be the preferred choice owing to resistance. Considering the resistance rates to oral antibiotics, trimethoprim/sulfamethoxazole may be a viable option because of its higher susceptibility to oral antibiotics.

The susceptibility of *P. aeruginosa* to ceftazidime was 50%, whereas resistance to carbapenems was observed in over 50% of the cases. This is more than twice the figure from domestic data from 2017 to 2019, where 22.1% exhibited carbapenem resistance [15].

Although *A. baumannii* had a lower frequency of isolation, the fact that over 90% of the isolated strains showed carbapenem resistance in culture was significant. This result aligns closely with that of Kor-GLASS, which reported a carbapenem resistance rate of 91.2% for *A. baumannii* [15]. Therefore, even if antibiotic susceptibility results are not available, carbapenem resistance should be considered when *A. baumannii* is identified in the culture at a clinically relevant frequency.

Globally, there is a trend of decreasing MRSA infections [21]. We also observed a similar trend in the methicillin resistance rates of *S. aureus* and CoNS in clinical specimens, consistent with findings from domestic Kor-GLASS data. The exact reasons for this decrease are not clear. However, one contributing factor could be the strengthening of infection control measures worldwide and decolonization [22, 23]. Additionally, changes in the characteristics of prevalent MRSA clones may play a role in this decrease [24].

The prevalence of CRE, particularly CPE, is low among patients transferred from LTCFs in Korea [6]. In another CRE surveillance study, no CRE cases were identified [7]. This raises questions regarding the cost-effectiveness of implementing active surveillance for CRE. However, the number of reported CRE cases has been increasing annually in South Korea [25]. The findings of this study further emphasize the increasing trend in CRE colonization rates over the years, starting at 1.7% in 2017 and reaching 20% by 2022. CPE has become more prevalent in South Korea, reaching approximately 61.9% by 2020 [26]. In this study, CPE accounted for 75.9% of all CRE cases. This significant increase underscores the importance of ongoing active surveillance of CRE colonization in patients transferred from LTCFs. Isolating patients with potential for contagion can be helpful in preventing the spread of infections. However, in Korea, hospital rooms are typically shared, making it difficult to isolate most patients transferred from LTCFs owing to factors such as being bedridden. Therefore, implementing individual isolation rooms for all patients could be restricted. Hence, the emphasis on horizontal precautions becomes crucial. Until the results are available, it should be emphasized to exercise caution and follow infection control measures, especially for potential CRE colonizers.

This study had several limitations. First, this was a single-center, retrospective investigation. Second, all results from cultures conducted at the time of admission were analyzed, regardless of whether infection was suspected. Although it is not possible to definitively attribute cultured organisms as the cause of infection, these cultures are typically conducted in patients with fever or suspected infection. Hence, it is reasonable to consider these cultured organisms as potential pathogens. Even if they are not the causative pathogens of infections, the presence of these organisms indicates the risk of future infection, making it valuable to assess their resistance profiles for clinical decision-making. This study provides the latest information on antibiotic resistance rates among LTCF referrals to a single hospital in South Korea; therefore, nationwide research is needed in the future.

## Conclusions

Patients transferred from LTCFs were mostly affected by infections, predominantly due to *Enterobacterales*. Owing to the high resistance rates, caution is necessary when selecting the initial antibiotics for infections. The increasing trend in CRE colonization rates each year highlights the need for the implementation of rigorous infection control measures for effective management.

### Supplementary Information

**Additional file 1: Supplementary Fig. 1. **Trends of antibiotics resistance. Further analysis of antimicrobial resistance trend are shown.

## Data Availability

The datasets generated and analyzed in the current study are available from the corresponding author upon reasonable request.

## Abbreviations

CCI: Charlson’s comorbidity index
CLSI: Clinical and Laboratory Standards Institute
CoNS: Coagulase-negative *Staphylococcus species*
COVID-19: Coronavirus disease
CPE: Carbapenemase-producing *Enterobacterales*
CRAB: Carbapenem-resistant *Acinetobacter baumannii*
CRE: Carbapenem-resistant *Enterobacterales*
EMRs: Electronic medical records
ESBLs: Extended-spectrum beta-lactamases
GI: Gastrointestinal
IQR: Interquartile range
LTCFs: Long-term care facilities
MICs: Minimal inhibitory concentrations
MRSA: Methicillin-resistant *Staphylococcus aureus*
UTIs: Urinary tract infections
