# Supplementary material for: Trends of antimicrobial susceptibilities and multidrug-resistant colonization rate in patients transferred from long-term care facilities during 2017–2022: a cross-sectional study
Source: BMC Infect Dis. 2024 Feb 21;24:235. doi: 10.1186/s12879-024-09145-y (PMC10882737; doi:10.1186/s12879-024-09145-y)
Supplement: Supplementary file 1 — Additional file 1: Supplementary Fig. 1. Trends of antibiotics resistance. Further analysis of antimicrobial resistance trend are shown. [file 12879_2024_9145_MOESM1_ESM.docx]

Supplementary Figure 1 Trends of antibiotic resistance. The rate of carbapenem resistance in (a) *Pseudomonas aeruginosa* and (b) *Acinetobacter baumannii* and (c) methicillin-resistant in *Staphylococcus species*.

(A)

*P<0.001*

(B)

*P=0.189*

(C)

*P=0.005*
